# Catastrophic Embolism Following Cosmetic Injection of Autologous Fat: Are Silicone-Treated Syringes the Only Suspects on the Crime Scene?

**DOI:** 10.3389/fsurg.2022.867994

**Published:** 2022-05-09

**Authors:** Aristo Vojdani, Yehuda Yulius Shoenfeld

**Affiliations:** ^1^ Immunosciences Lab. Inc., Los Angeles, CA, United States; ^2^ Cyrex Labs LLC, Phoeniz, AZ, United States; ^3^ Ariel University, Ariel, Samaria, Israel; ^4^ Zabludowicz Center for Autoimmune Diseases, Sheba Medical Center, Tel-Aviv University, Ramat Gan, Israel

**Keywords:** silicone_1_, syringe_2_, AFI_3_, SiOPs_4_, embolism_5_, POPs_5_

## Introduction

The use of silicone-lubricated syringes for autologous fat injection [AFI] has become very popular in the fields of dermatology and plastic surgery. It has been used successfully in facial rejuvenation, scar contracture repair, and more (1, 2). This procedure aims to improve deep defects in the skin surface through injections of the patient’s own separated fatty tissue (3). AFI is considered by many practitioners as a minimally invasive and safe procedure, with possible complications such as fat necrosis, oil cyst formation, calcification, and infection (4). However, it should be noted that in addition to these non-fatal complications, there have been reports of several cases of fatal cerebral fat embolism and stroke. For example, the injection of fat into the facial/eye orbit may cause a reflux of fat into the ophthalmic or cerebral arteries, resulting not only in fat embolism, but, in very rare cases, even blindness or cerebral infarction (5–8). Although these rare but devastating consequences of what was then called autologous fat grafting [AFG] have been in the literature since the 1980s, two striking cases that were published very recently by Lieu et al. in *Frontiers in Medicine* have rekindled interest in the possibility of catastrophic embolism following facial AFI (9). The article describes how two women respectively received AFI into the temporal and frontal areas. The first woman underwent decompressive craniectomy, suffered a continuous deterioration of her condition, and died. Imaging examinations showed occlusion of the right external carotid artery. The second woman’s vision had failed to improve at the 3-month follow-up visit. Her examinations showed that multiple retinal arterioles were segmentally occluded. Liu et al. also searched reference lists and identified a total of 26 articles that dealt with similar topics. They concluded that since AFI in healthy adults can lead to such devastating and even fatal complications, the procedure ‘should be performed gently and slowly with low pressure, and blunt needles may be the most appropriate instruments’ (9). The potential mechanisms listed for this cerebral infarction included cerebral artery injury, increased blood coagulation, decreased blood supply, air or fat embolism, and emboli from the aorta. However, the Liu group did not discuss whether additional factors within fat tissue and the syringes used in the process could be responsible for this AFI-induced catastrophic embolism.

## Silicone Oil and Its Adjuvant Activity

This possibility was investigated by other research teams who connected these terrible complications of fat injection to the use of silicone oil as a lubricant to coat the barrel of the syringe in order to make it easier to move the plunger with reasonable pressure during the injection (9–15). These syringes are used to inject fat cells, insulin, allergens, vaccines, antigens, monoclonal antibodies (including SARS-CoV-2 spike protein antibodies), anti-vascular endothelial growth factor, anti-cytokines, and more, and due to this lubrication process, thousands, if not millions, of silicone oil droplets are mixed in with the different contents of these syringes (15, 16). It is not unreasonable to posit some consequence from the injection of these silicone oil droplets into our systems. One good example might be the appearance of silicone floaters in the eye as a result of Avastin eye injections. Because silicone is known to be an excellent adjuvant (17), the pharmaceutical industry and regulatory agencies have serious concerns that silicone oil particles (SiOPs) have the potential to increase protein aggregates, or, that by functioning as adjuvants, they might generate anti-protein or anti-haptenic chemical antibodies (18–21). In fact, various recombinant proteins have been shown to bind to SiOPs released from emulsified silicone oil which is usually included in the proteins used as therapeutics (18, 20, 22). For example, IgG_1_ monoclonal antibody is capable of effector function; if this protein forms aggregates with silicone droplets, the resulting aggregate may mimic immune complexes that trigger antibody-dependent cell-mediated cytotoxicity, which is involved in type II allergic reactions (23). Furthermore, SiOPs-adsorbed antigens may mimic pathogen-associated molecular patterns (PAMPs), which may possibly result in enhanced phagocytosis, cytokine release, and autoinflammatory reaction (24, 25).

The adjuvant activity, enhanced phagocytosis, and induction of protein aggregates by silicone is well-known for the role of all of these in the development of multi-organ system disorders, including rheumatic and neurologic diseases. There are many articles and case reports from the time period 1983–1995 alone (26–47) that address this.

In one of my own articles from back then, my group hypothesized that an immune reaction to silicone breast implants would include host reactivity not just against the silicone but also the macromolecules within the environment of the implant, and that the generated autoantibodies may react with other tissue antigens distal from the site of the implant (46). To test this hypothesis, we obtained sera from 520 symptomatic women with silicone implants who had developed silicone-related immunological disorders, and also from 520 matched controls without implants. We tested these sera for the presence of antibodies against silicone bound to human serum albumin (HSA), HSA alone, and myelin basic protein (MBP). Antibodies against these antigens were detected in about 2% of healthy controls, and in up to 43% of the symptomatic patients with implants.

Based on our findings and the literature available at the time (26–47), we concluded that the silicone breast implant oozes or bleeds small silicone particles, which are then absorbed or bound to macromolecules surrounding the silicone bag. The tissue antigens bound to silicone spheres are presented to macrophages. Cooperation between T-helper cells and B cells produce specific antibodies, which may react specifically to both the silicone and the tissues from which the tissue antigens bound to the silicone originated. You may see Supplementary Figure S1 (in Supplementary Material), which shows the original illustration from our 1994 article; it summarizes the hypothetical mechanism described above and shows how silicone can contribute to autoimmunity.

## Possible Association between Silicone Implants and Autoimmunity

It is worth noting that these articles we have cited about silicone breast implants and their association with many autoimmune disorders were published before the introduction of autoimmune/inflammatory syndrome induced by adjuvants (Shoenfeld’s syndrome) in 2011 (48). In fact, in 1994 I and two of my colleagues collaborated to write an editorial titled ‘Silicone breast implants and autoimmunity: Causation or myth?’ (45). Studies like the ones we have cited, together with major public outcry, led the Food and Drug Administration (FDA) to ban the use of silicone breast implants for cosmetic purposes in 1992. However, the pressing demand for cosmetic breast implants led to the introduction of saline and other types of breast implants. Today there are four general types of breast implants defined by their filler material: silicone gel, saline solution, structured, and composite filler. No matter what the filler is, the implants use sacs that are still elastomer silicone shells. All of these types of implants are currently on the market and freely available for cosmetic augmentation, *including silicone breast implants*, because in 2006 the US government ended the 14-year ban on them. A look at the FDA website today will simply reveal general warnings about all types of breast implants. However, there soon came a rising tide of increasing reports not only about specific autoimmune diseases but also autoimmune-related or peripheral disorders that could not easily be categorized. This is what led Shoenfeld and Agmon-Levin to introduce autoimmune/inflammatory syndrome induced by adjuvants, which would later come to be called Shoenfeld’s syndrome (48). In this article published in the *Journal of Autoimmunity*, the authors found that adjuvants, which are factors normally used to activate or enhance immune response for a positive or beneficial purpose, could also be associated with both defined and non-defined immune-mediated diseases. One of these conditions was siliconosis, with the adjuvant being silicone.

With this kind of history behind silicone and its association with ASIA or Shoenfeld’s syndrome, we should not be surprised that silicone-treated syringes may have a role in the induction of catastrophic embolism, coagulopathy, and anti-phospholipid syndrome after autologous fat injection, as already discussed above. However, we believe that silicone-treated syringes may not be the only perpetrators of this crime of inducing autoimmunity.

Silicone in the syringes may not be the only perpetrators of autoimmunity induction. To find other suspects, we turn from the silicone in the syringe to the actual fat or adipose tissue used in AFI. In reviewing the structure of the fat cell or adipocyte, we note that like any other cell it contains a cell membrane, cytoplasm, Golgi apparatus, nucleus, mitochondrion, and more. However, a very large portion of the adipocyte’s cytoplasm is occupied by the fat reservoir. This fat reservoir serves as the storage compartment for lipophilic persistent organic pollutants (POPs). The size of these cells depends on the amount of chemicals stored within them. Due to their molecular structure, POPs can bind to the membrane, particularly to the phospholipids, endoplasmic reticulum, and even to the mitochondrion. Overall, the accumulation of pollutants, especially lipophilic POPs, in adipocytes increases the total body burden of toxic chemicals (49–51).

Here’s what you should know about POPs:

•  Highly toxic chemicals
  - Pesticides
  - Industrial chemicals
  - Unwanted industrial by-products harmful to humans and the environment
•  An estimated 400 million tons are produced annually worldwide
•  The dirty dozen: aldrin, chlordane, DDT, dieldrin, dioxins, furans, endrin, HCB, heptachlor, mirex, PCBs, toxaphene
•  Stored in fat, persistent
•  Each one of us, depending on our body weight and percentage of adipose tissue, may carry a few grams of each of the above chemicals in our fat
•  The main routes of exposure to these toxic chemicals are seafood (salmon, eel, shellfish, fish liver, fish oil), animal fat (meat, poultry), cow’s milk (butter, dairy products) and other foods such as vegetables, cereals and fruit.

## Discussion

**Figure 1:**
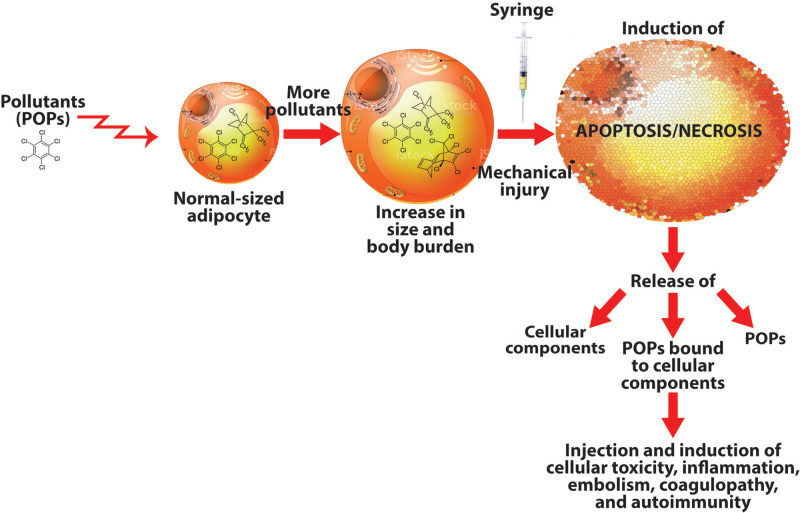
How pollutants stored in adipocytes may lead to autoimmunity. Adipocytes filled with stored persistent organic pollutants (POPs) may burst upon extraction, releasing the POPs, cellular components, and POPs bound to cellular components into the syringe, which will inject all these materials into the patient, possibly inducing immune-mediated disorders.

Considering that various interactions between adipose tissue and POPs have been reported (52, 53), and increased exposure to these toxic chemicals results in greater body burden within the liver and particularly in adipocytes, any interference with the physiological environment of adipocytes may result in the release of these pollutants into the environment of the tissue, and/or, the adipocytes may undergo apoptosis or necrosis (see Figure 1). How does all this relate to autologous fat injection?

The collection of fat tissue using syringe, mechanical or enzymatic treatment may result in the induction of the apoptosis/necrosis program in some adipocytes. When these cells die/disintegrate, they will release their cellular contents (including the POPs and POPs bound to the adipocyte’s cellular components) into the syringe. The injection of these factors released from damaged adipocytes along with many healthy cells into genetically susceptible individuals may result in fat embolism, coagulopathy, and autoimmune reactivity against various autoantigens. It behooves us, then, whether patient or practitioner, that whenever we see someone poised with a syringe in hand about to inject its contents into a living human being, we should reflect carefully on the many items that may be included in those contents. You may see Supplementary Figure S2 (in Supplementary Material), which shows some of these contents related to this matter.

While we do not have solid experimental evidence for the contributions of POPs in adipocytes to the development of these disorders, we hope that our work will generate further investigation towards identifying other suspects aside from silicone-treated syringes for the crime of specific and non-specific immune disorders through the modus operandus of autologous fat injection.
