# Supplementary figures and images for: Catastrophic Embolism Following Cosmetic Injection of Autologous Fat: Are Silicone-Treated Syringes the Only Suspects on the Crime Scene?
Source: Front Surg. 2022 May 9;9:867994. doi: 10.3389/fsurg.2022.867994 (PMC9124850; doi:10.3389/fsurg.2022.867994)

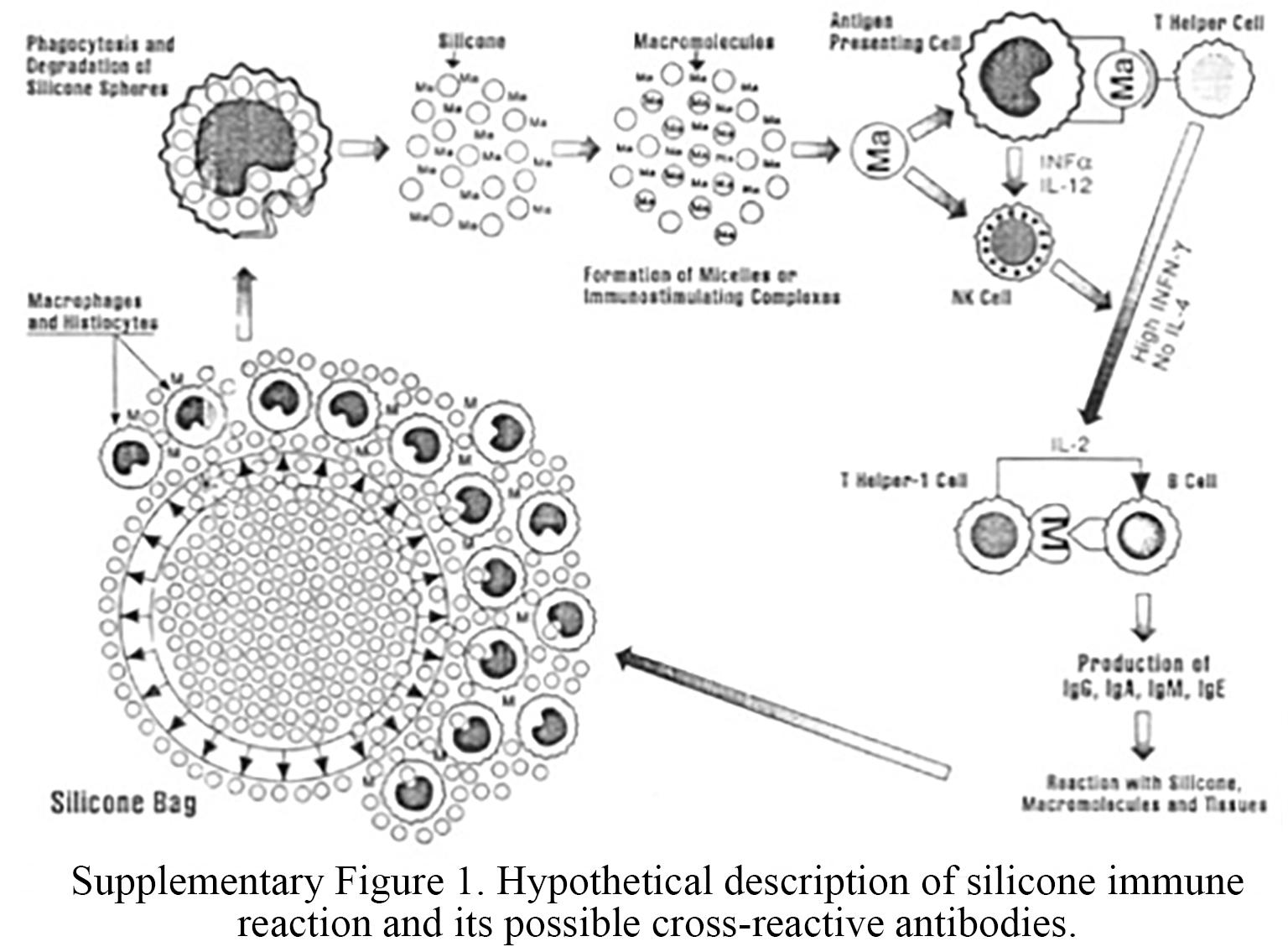

Supplement: Supplementary file 1 [file fsurg2022867994_Image_1_v1.JPEG]

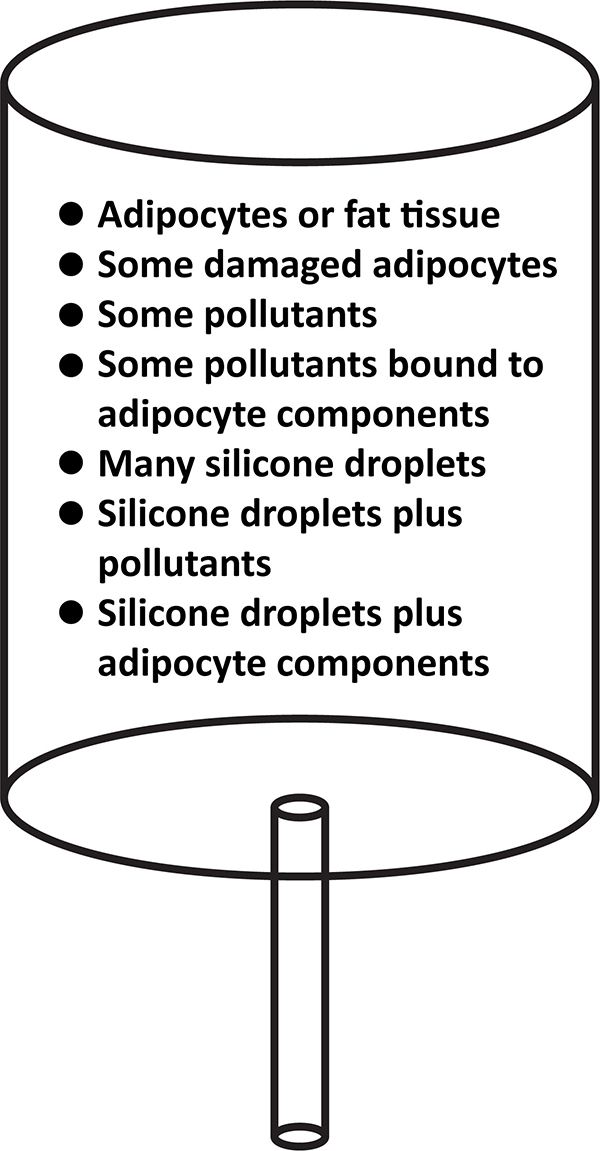

Supplement: Supplementary file 2 [file fsurg2022867994_Image_2_v1.JPEG]
